# Association of skeletal muscle mass and its change with diabetes occurrence: a population-based cohort study

**DOI:** 10.1186/s13098-023-01027-8

**Published:** 2023-03-22

**Authors:** Yiting Xu, Tingting Hu, Yun Shen, Yufei Wang, Yuqian Bao, Xiaojing Ma

**Affiliations:** Department of Endocrinology and Metabolism, Sixth People’s Hospital Affiliated to Shanghai JiaoTong University School of Medicine; Shanghai Clinical Center for Diabetes; Shanghai Diabetes Institute; Shanghai Key Laboratory of Diabetes Mellitus, 600 Yishan Road, Shanghai, 200233 China

**Keywords:** Skeletal muscle mass, Diabetes occurrence, Sarcopenia, Muscle mass loss, Impaired glucose regulation

## Abstract

**Background:**

Low muscle mass likely results in reduced capacity for glucose disposal, leading to a significant but under-appreciated contribution to increasing the risk of diabetes. But few prospective studies have investigated the association between the loss of muscle mass and the occurrence of diabetes. We aimed to investigate whether short-term changes in muscle mass affect the incidence of diabetes in a Chinese population.

**Methods:**

This study included 1275 individuals without evident diabetes at baseline. In the baseline and re-examination, individuals completed the risk factors survey and underwent body composition measurement. Muscle mass index was defined as the percentage skeletal muscle mass, which was measured by an automatic bioelectric analyzer.

**Results:**

After a median follow-up of 2.1 years, 142 individuals developed diabetes (11.1%). There was an inverse association between basal skeletal muscle mass index and the risk of diabetes in participants with impaired glucose regulation but not in those with normal glucose tolerance. Multivariate-adjusted hazard ratios for the risk of developing diabetes were 0.85 (95% CI: 0.74–0.98) and 1.15 (95% CI: 0.98–1.34), respectively. Furthermore, Cox regression analysis revealed that a two-year change in skeletal muscle mass was also inversely associated with the incidence of diabetes in both participants with normal glucose tolerance and with impaired glucose regulation (HR: 0.76, 95% CI: 0.65–0.89; HR: 0.81, 95% CI: 0.71–0.91).

**Conclusions:**

These findings emphasized the importance of early detection and control of muscle mass loss for the prevention of diabetes.

## Background

The global prevalence of diabetes continues to increase rapidly, with 537 million people diagnosed worldwide in 2021. This number is projected to increase by 46%, reaching 783.2 million by 2045 [1, 2]. Part of the increase in diabetes incidence results from increasing prevalence of metabolic risk factors which are important drivers of diabetes development; therefore, it is of great importance to identify modifiable factors for the prevention of diabetes.

Sarcopenia, described as a multidimensional condition requiring assessment of muscle mass, muscle strength, and physical performance [3–6], may have a significant but under-appreciated contribution to increasing the risk of diabetes [7–9]. Given that skeletal muscle is the largest insulin-sensitive tissue in the body and accounts for 80% of glucose uptake under euglycemic and hyperinsulinemic conditions, low muscle mass likely results in reduced capacity for glucose disposal. However, the metabolic outcomes of sarcopenia have received less attention than the functional consequences in research literature. One study has reported that low muscle mass was associated with an increased risk of type 2 diabetes [10]; beyond that, few prospective studies have regarded this issue, and no prospective studies have investigated the association between change in muscle mass and the occurrence of diabetes.

To fill these knowledge gaps, we aimed to prospectively examine the association of muscle mass change with an increased risk of diabetes in a community-based population.

## Methods

### Study population

The present study is a prospective, population-based cohort study in community-dwelling ambulatory adults aged over 20 years. Eligible participants were recruited through promotional posters in a local community health center in Shanghai. An original cohort of 2433 participants was enrolled in 2013–2014. All the participants gave their informed consent and underwent standardized health assessments for demographic data, lifestyle habits, medical history, laboratory testing, and body composition measurement. In 2015–2016, these participants were invited for a second examination that was similar to the previous. The current study was carried out in accordance with the principles of the Declaration of Helsinki and approved by the institutional review board at the Ethics Committee of Sixth People’s Hospital Affiliated to Shanghai Jiao Tong University School of Medicine.

For this study, we included 1277 participants who were non-diabetic at baseline, completed the 2015–2016 examination, and with fasting and 2-h blood specimens and body composition data available. Of those participants, we excluded two with inadequate serum samples, resulting in a final analysis of 1275 participants.

### Glucose and body composition measurement

Venous blood specimens were collected during a morning visit after a 10-h overnight fast. Participants also provided a 2-h plasma glucose specimen following a 75-g oral glucose tolerance test. Glucose, fasting insulin, and lipid profiles were analyzed using Hitachi 7600–120 automatic biochemical analyzer (Hitachi, Tokyo, Japan). Fasting and 2-h plasma glucose specimens were measured using the standard glucose oxidase method, and fasting insulin was measured using the electrochemiluminescence immunoassay method. Total cholesterol and triglyceride were measured via enzymatic procedures, and high-density lipoprotein cholesterol and low-density lipoprotein cholesterol were measured via direct assay method.

Total body fat percentage was measured using an automatic bioelectric analyzer (BIA; TBF-418B; Tanita Corp., Tokyo, Japan). Skeletal muscle mass was calculated using the BIA equation of Janssen et al*.*: skeletal muscle mass (kg) = [(height^2^/BIA-resistance × 0.401) + (sex × 3.825) + (age × –0.071)] + 5.102, where height is in cm; BIA-resistance is in ohms; sex, men = 1 and women = 0; and age is in years. Skeletal muscle index was calculated by the conversion of absolute skeletal muscle mass (kg) to percentage skeletal muscle mass using the formula: muscle mass/body mass × 100. Relative muscle mass change was calculated by subtracting muscle mass index at baseline from measured muscle mass index at re-examination, divided by muscle mass index at baseline, multiplied by 100%.

### Definition of diabetes

At baseline and subsequent follow-up examinations, diabetes was identified by self-report, the use of oral hypoglycemic agents and/or insulin, or oral glucose tolerance test with a fasting plasma glucose concentration of ≥ 7.0 mmol/l or a post-load glucose concentration of ≥ 11.1 mmol/l after a 75 g oral glucose tolerance test, or a glycated hemoglobin A1c concentration of ≥ 6.5%. Impaired glucose regulation was defined as fasting plasma glucose between 6.1 and 7.0 mmol/l, and/or 2-h plasma glucose between 7.8 and 11.1 mmol/l [11].

### Assessment of main covariates

All covariates included in this study were collected at baseline and re-examination. Information on age, sex, smoking status, physical activity, disease status, and medication use was collected from interviews using standardized questionnaires. Body weight, height, and blood pressure were obtained during physical examinations at a local community health center. BMI was calculated as weight in kilograms divided by height in meters squared. Homeostasis model assessment was used to estimate insulin resistance (HOMA-IR) and was calculated as fasting plasma glucose (mmol/L) × fasting insulin (mU/L)/22.5. Smoking was defined as the use of at least one cigarette per day for at least 6 months. Alcohol use was defined as the consumption of at least 20 g of alcohol per day for at least six months. Level of physical activity was classified as light, moderate, and high according to the 2001 International Physical Activity Questionnaire [12]. A dietary quality score was defined according to 5 healthy dietary behaviors collected by the food frequency questionnaire, including a high intake of fruits and vegetables (over 4.5 cups per day), fish (over two 3.5 oz servings per week), and soy food (over 25 g per day), and a low intake of sweetened beverages (less than 450 kcal per week) and red meat (less than 50 g per day). Each variable was scored as 0 or 1, and the total score was summed up individually ranging from 0 to 5. A higher score indicated a healthier diet [13].

### Statistical analysis

Continuous data were presented as mean (SD) or median (interquartile range) according to whether the variable was normally distributed. Categorical variables were described as number (proportion). Paired-Samples *t* test and Wilcoxon test were used to assess differences between baseline and re-examination for continuous variables, respectively, and Chi square test was used for categorical variables. Linear regression was applied to examine the associations between the change in skeletal muscle index and change in glucose, insulin, and HOMA-IR levels. Restricted cubic spline regression with three knots (5th, 50th, and 95th) was used to examine a dose–response relationship between the change in skeletal muscle index and the risk of newly diagnosed diabetes. Relative muscle mass change was classified as 4 categories: ⊿skeletal muscle mass index loss of more than 8%, change between − 8% and < –2%, change between − 2% and < 2% (muscle mass maintenance: reference category), and ≥ 2%. The Cox proportional hazards model was used to estimate hazard ratios (HRs) and 95% confidence interval (CI) for the association of baseline skeletal muscle index and its change with the risk for diabetes incidence in participants with normal glucose tolerance or impaired glucose regulation. In the multivariate models, we adjusted for age, sex, systolic blood pressure, diastolic blood pressure, triglyceride, high-density lipoprotein cholesterol, low-density lipoprotein cholesterol, smoking, alcohol consumption, dietary quality score, physical activity, and total body fat percentage. Age-, sex-, BMI-, and baseline skeletal muscle index-stratified analyses were conducted to evaluate whether the change in skeletal muscle mass index was associated with the occurrence of diabetes.

Restricted cubic spline regression analyses were performed using the R software package, version 4.0.3 (R Foundation for Statistical Computing, Institute for Statistics and Mathematics, Wien, Austria); other statistical analyses were performed using IBM SPSS Statistics for Windows, version 20.0 (SPSS Inc., Chicago, NC, USA). Statistical significance was set at *P* < 0.05 (two-tailed).

## Results

### Characteristics at baseline and at re-examination

A total of 1275 individuals, 496 men and 779 women, with an age range of 24 to 76 (mean 57.7 ± 7.03) years, were enrolled in this study. After a median follow-up of 2.1 years, 142 (11.1%) individuals had developed diabetes; among the population, 53 (6.1%) individuals had developed diabetes in those with normal glucose tolerance and 89 (21.9%) individuals had developed diabetes in those with impaired glucose tolerance. General characteristics of the study population at baseline and re-examination are given in Table 1. Mean BMI, fasting plasma glucose, glycated hemoglobin A1c, total cholesterol, triglyceride, high-density lipoprotein cholesterol, and low-density lipoprotein cholesterol were all significantly higher at re-examination than at baseline, whereas mean diastolic blood pressure was lower in participants with normal glucose tolerance or impaired glucose regulation (*P* < 0.01). Among the participants with normal glucose tolerance, the mean 2-h plasma glucose was higher (*P* < 0.01), and the proportions of smoking and high physical activity were lower (*P* < 0.05) at re-examination. There were no significant differences in skeletal muscle mass change and relative skeletal muscle mass change between normal glucose tolerance and impaired glucose regulation groups (both *P* > 0.05).

**Table 1:** Characteristics of study participants at baseline and at re-examination

Characteristics	Normal glucose tolerance (n = 868)		Impaired glucose regulation (n = 407)	
	Baseline	Re-examination	Baseline	Re-examination
Men, n (%)	319 (36.8)		177 (43.5)	
Age, years	57.0 ± 7.04	58.9 ± 7.07^**^	59.1 ± 6.80	61.1 ± 6.83^**^
Body mass index, kg/m^2^	23.7 ± 3.17	24.0 ± 3.31^**^	24.3 ± 2.94	24.5 ± 2.98^**^
Total body fat percentage, %	27.4 ± 8.18	27.5 ± 8.35	27.6 ± 7.97	27.6 ± 8.11
Skeletal muscle mass index, %	32.0 ± 5.57	31.8 ± 5.70^*^	32.1 ± 5.51	32.0 ± 5.57
Blood pressure, mmHg				
Systolic	128 (118–140)	129 (117–141)	130 (120–144)	132 (122–145)
Diastolic	79.0 (72.0–85.3)	77.0 (70.0–84.0)^**^	80.0 (73.0–87.0)	78.0 (72.0–86.0)^**^
Fasting plasma glucose, mmol/L	5.12 ± 0.44	5.66 ± 0.56^**^	5.35 ± 0.64	6.07 ± 0.72^**^
2-h plasma glucose, mmol/L	6.05 ± 1.09	6.82 ± 1.91^**^	8.81 ± 1.23	8.64 ± 2.39
Glycated hemoglobin A1c, %	5.50 ± 0.33	5.65 ± 0.40^**^	5.67 ± 0.35	5.81 ± 0.42^**^
Total cholesterol, mmol/L	5.13 ± 0.90	5.40 ± 1.02^**^	5.10 ± 0.89	5.38 ± 0.97^**^
Triglyceride, mmol/L	1.24 (0.87–1.76)	1.33 (0.95–1.93)^**^	1.46 (0.97–2.04)	1.52 (1.10–2.23)^**^
High-density lipoprotein cholesterol, mmol/L	1.39 ± 0.35	1.45 ± 0.36^**^	1.32 ± 0.33	1.38 ± 0.34^**^
Low-density lipoprotein cholesterol, mmol/L	3.11 ± 0.77	3.26 ± 0.87^**^	3.13 ± 0.75	3.25 ± 0.81^**^
Smoking, n (%)	175 (20.2)	161 (18.5)^*^	76 (18.7)	76 (18.7)
Alcohol use, n (%)	94 (10.8)	88 (10.1)	43 (10.6)	44 (10.8)
Physical activity, n (%)				
Light	157 (18.1)	163 (18.8)^*^	69 (17.0)	62 (15.2)
Moderate	386 (44.5)	437 (50.3)^*^	212 (52.1)	237 (58.3)
High	325 (37.4)	268 (30.9)^*^	126 (30.9)	108 (26.5)
Skeletal muscle mass change, kg	− 0.13 (− 1.35–0.97)		− 0.09 (− 1.39–1.09)	
Skeletal muscle mass index change, %	− 0.44 (− 4.37–3.12)		− 0.33 (− 4.42–3.40)	

^*^P < 0.05, ^**^P < 0.01

### Associations of change in skeletal muscle mass index and changes in glucose

**Fig. 1:**
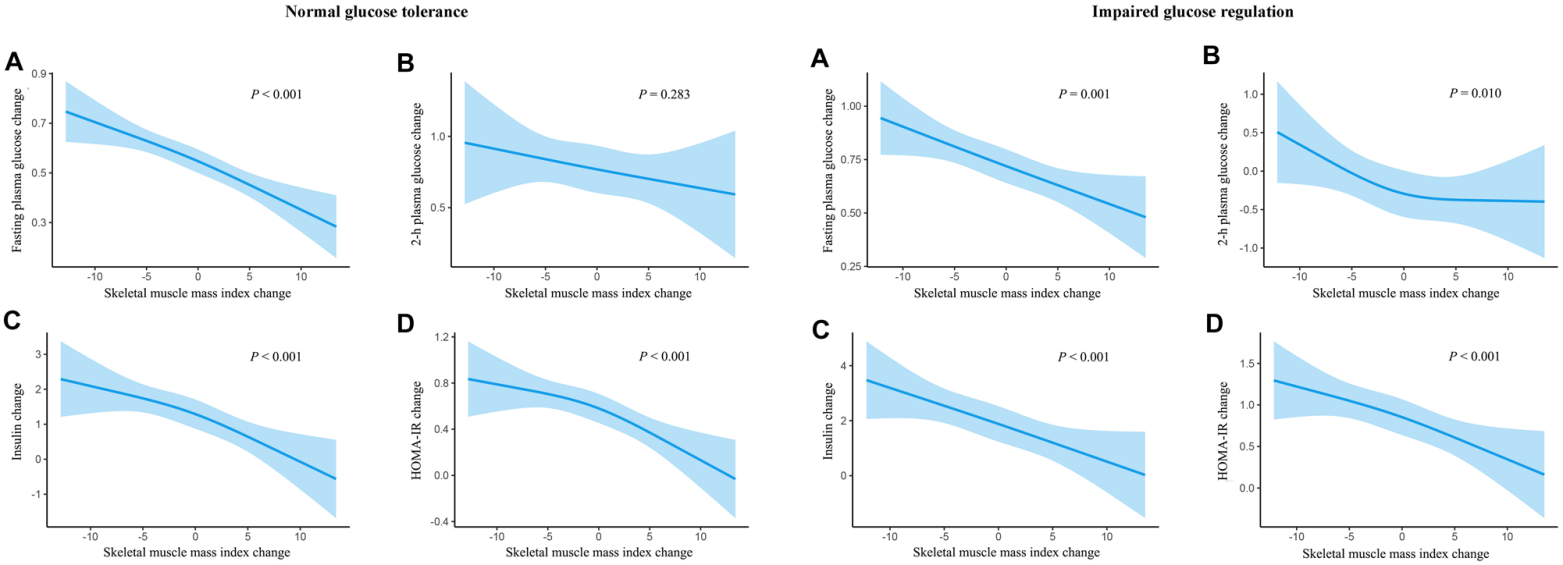
Associations of change in skeletal muscle mass index and changes in glucose parameters (**A** fasting plasma glucose change;** B** 2-h plasma glucose change;** C** insulin change;** D** HOMA-IR change) according to different glucose tolerance status

As shown in Fig. 1, a decreasing trend of changes in fasting plasma glucose, fasting insulin, and homeostasis model assessment-insulin resistance index went along with an increasing change of skeletal muscle mass index after adjusted for age, sex and baseline skeletal muscle mass index, regardless of glucose tolerance status (all* P* < 0.05). Moreover, there was a significantly inverse association between the change of skeletal muscle mass index and the change of 2-h plasma glucose in the impaired glucose regulation group (*P* < 0.010), but the association was non-significant in the normal glucose tolerance group (*P* = 0.283).

## Change in skeletal muscle mass index and diabetes occurrence in normal glucose tolerance and impaired glucose regulation

**Fig. 2:**
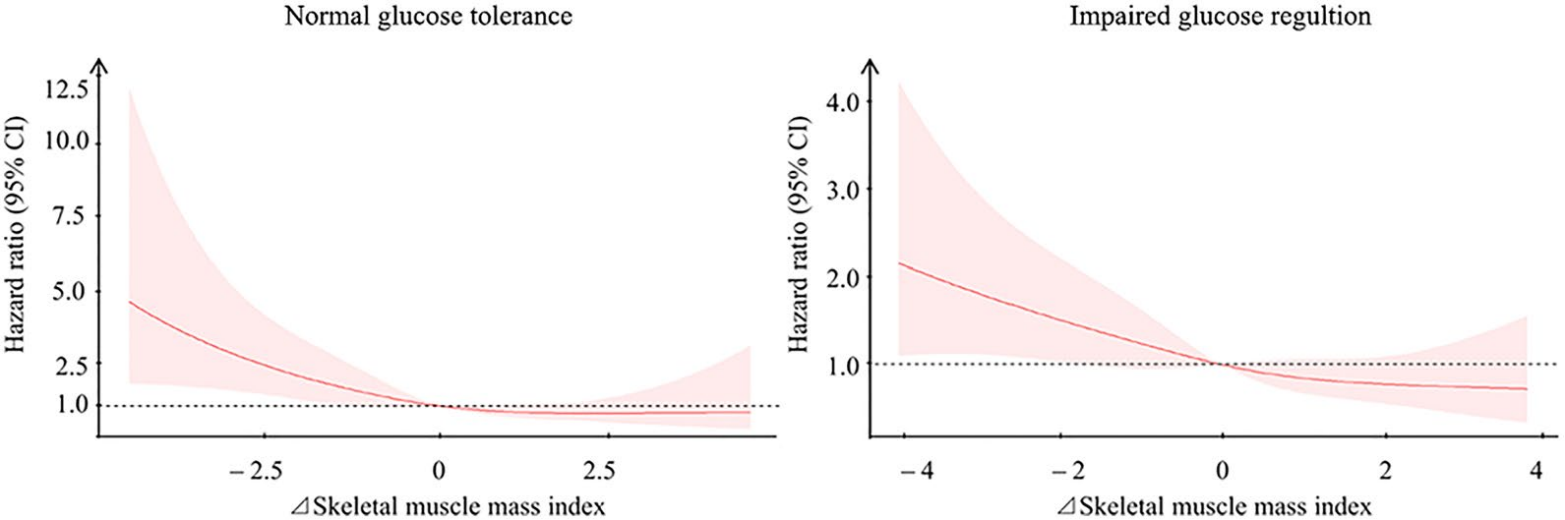
Association between change in skeletal muscle mass index and the occurrence of diabetes in participants with normal glucose tolerance and impaired glucose regulation

**Fig. 3:**
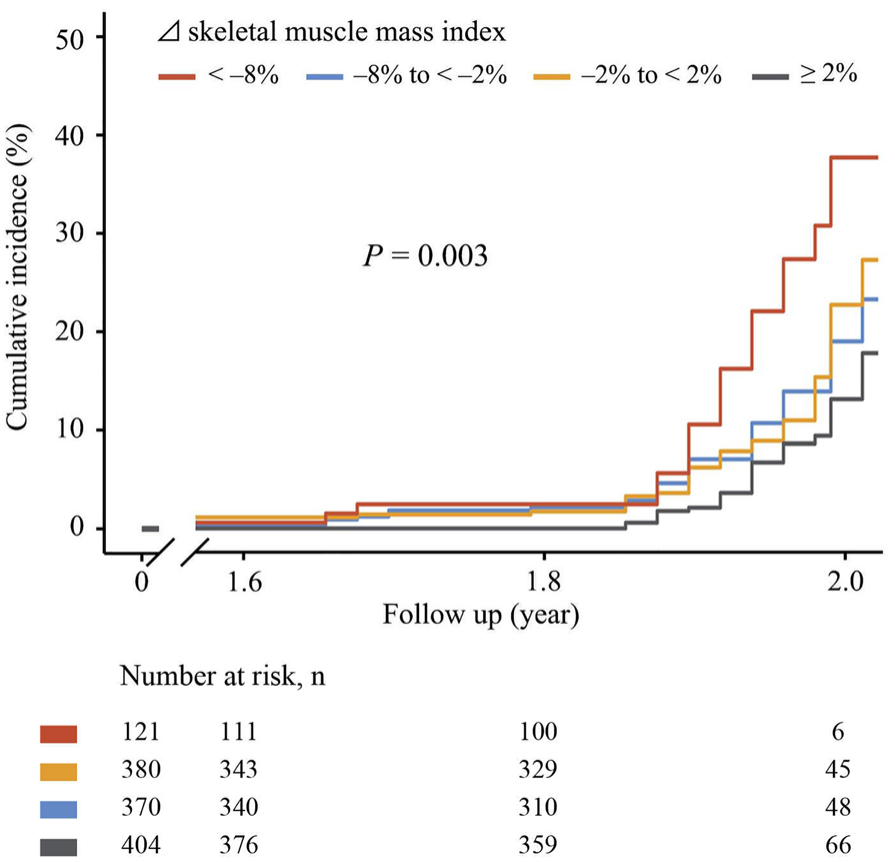
Kaplan–Meier survival analysis of skeletal muscle mass index changes for cardiovascular events

Restricted cubic spline models showed that a decrease in skeletal muscle mass index was associated with a nearly 2-fold risk of developing diabetes in both participants with normal glucose tolerance and impaired glucose regulation (Fig. 2). Kaplan–Meier analysis was further used to determine the association between skeletal muscle mass index change categories and cardiovascular events risk (Fig. 3). The results showed that participants with a relative muscle mass loss over 8% had the highest cumulative incidence of cardiovascular events, while those with a relative skeletal muscle mass gain ≥ 2% had the lowest cumulative incidence of cardiovascular events. Among the participants with normal glucose tolerance, Cox regression analyses showed that those with a relative muscle mass loss of more than 8% had a 2.58-fold (95% CI: 1.12–5.96) risk of diabetes compared with those who had stable muscle mass; besides, those with a relative muscle mass change ≥ 2% had 63% decreased risk of diabetes compared with those with stable muscle mass (Table 2). Increasing continuous skeletal muscle mass index change was associated with a lower risk of diabetes incidence after multivariate adjustment (HR: 0.76, 95% CI: 0.65–0.89; Table 2). Participants with impaired glucose regulation and with a relative muscle mass loss of more than 8% had a 2.44-fold (95% CI: 1.11–5.35) risk of diabetes compared with those who had stable muscle mass. A 2-year change in continuous skeletal muscle mass index was significantly and inversely associated with the occurrence of diabetes (HR: 0.81, 95% CI: 0.71–0.91; Table 2).

**Table 2:** Multiple adjusted hazard ratio of diabetes by basal skeletal muscle mass index and its change among subjects with normal glucose tolerance or impaired glucose regulation

	Cases of diabetes	Pearson-years	Multiple adjusted hazard ratios (95% confidence intervals)	
			Model 1	Model 2
Total (n = 1275)				
Basal skeletal muscle mass index	142	2421	0.98 (0.93–1.04)	0.98 (0.89–1.09)
⊿ Skeletal muscle mass index categories				
< –8%	21	227	2.45 (1.42–4.23)^a^	2.65 (1.52–4.62)^a^
− 8% to < − 2%	44	721	1.05 (0.69–1.62)^a^	1.12 (0.72–1.72)^a^
− 2% to < 2%	42	698	Reference	Reference
≥ 2%	35	775	0.59 (0.38–0.93)^a^	0.60 (0.38–0.94)^a^
Continuous ⊿ skeletal muscle mass index	142	2421	0.81 (0.74–0.88)^a^	0.79 (0.72–0.87)^a^
Normal glucose tolerance (n = 868)				
Basal skeletal muscle mass index	53	1647	1.03 (0.94–1.13)	1.15 (0.98–1.34)
⊿ Skeletal muscle mass index categories				
< − 8%	10	146	2.64 (1.17–5.93)^a^	2.58 (1.12–5.96)^a^
− 8% to < − 2%	16	512	0.81 (0.40–1.61)^a^	0.79 (0.39–1.60)^a^
− 2% to < 2%	17	475	Reference	Reference
≥ 2%	10	514	0.37 (0.17–0.83)^a^	0.37 (0.17–0.82)^a^
Continuous ⊿ skeletal muscle mass index	53	1647	0.75 (0.65–0.88)^a^	0.76 (0.65–0.89)^a^
Impaired glucose regulation (n = 407)				
Basal skeletal muscle mass index	89	774	0.95 (0.87–1.03)	0.85 (0.74–0.98)
⊿ Skeletal muscle mass index categories				
< − 8%	11	80	1.90 (0.89–4.05)^a^	2.44 (1.11–5.35)^a^
− 8% to < − 2%	28	210	1.38 (0.79–2.41)^a^	1.64 (0.92–2.92)^a^
− 2% to < 2%	25	223	Reference	Reference
≥ 2%	25	261	0.78 (0.44–1.38)^a^	0.78 (0.43–1.40)^a^
Continuous ⊿ skeletal muscle mass index	89	774	0.85 (0.76–0.96)^a^	0.81 (0.71–0.91)^a^

Model 1 was adjusted for age, sex, systolic blood pressure, diastolic blood pressure, triglyceride, high-density lipoprotein cholesterol and low-density lipoprotein cholesterol. Model 2 was further adjusted for smoking, alcohol use, dietary quality score, physical activity, and total body fat percentage

^a^The model was further adjusted for basal skeletal muscle mass index

### Subgroup analyses

Additionally, we stratified participants’ age, sex, mean BMI, and mean baseline skeletal muscle mass index to control the potential confounders. In these subgroups, participants with a relative muscle mass loss of more than 8% had a 2 or 3 folds risk of developing diabetes compared with those who had stable muscle mass. The multivariate adjusted association between a 2-year change in skeletal muscle mass index and the risk of diabetes incidence remained statistically significant in stratified analyses (Table 3).

**Table 3:** Multiple adjusted hazard ratio for the incidence of diabetes by skeletal muscle mass index change in subgroups in total subjects

Subgroup	Multiple adjusted hazard ratios (95% confidence intervals)				
	⊿ skeletal muscle mass index categories				Continuous ⊿ skeletal muscle mass index
	< − 8%	− 8% to < –2%	− 2% to < 2%	≥ 2%	
Age					
< 60 years	2.16 (1.00–4.68)	0.79 (0.42–1.51)	Reference	0.56 (0.29–1.13)	0.78 (0.68–0.89)
≥ 60 years	3.08 (1.32–7.17)	1.66 (0.89–3.08)	Reference	0.65 (0.34–1.23)	0.79 (0.68–0.92)
Sex					
Men	2.38 (0.77–7.39)	1.24 (0.66–2.34)	Reference	0.45 (0.22–0.93)	0.80 (0.68–0.94)
Women	2.95 (1.49–5.86)	1.08 (0.58–1.99)	Reference	0.67 (0.36–1.26)	0.76 (0.67–0.86)
Body mass index					
< 25 kg/m^2^	2.36 (1.10–5.10)	1.28 (0.71–2.33)	Reference	0.75 (0.41–1.37)	0.84 (0.74–0.95)
≥ 25 kg/m^2^	3.21 (1.35–7.64)	0.90 (0.46–1.75)	Reference	0.44 (0.21–0.91)	0.76 (0.65–0.88)
Basal Skeletal muscle mass index					
< 32%	2.60 (1.27–5.32)	0.96 (0.52–1.77)	Reference	0.59 (0.32–1.07)	0.77 (0.67–0.89)
≥ 32%	3.74 (1.48–9.46)	1.37 (0.73–2.59)	Reference	0.53 (0.26–1.10)	0.76 (0.66–0.88)

The multiple adjusted model was adjusted for age, sex, systolic blood pressure, diastolic blood pressure, triglyceride, high-density lipoprotein cholesterol, low-density lipoprotein cholesterol, basal skeletal muscle mass index, smoking, alcohol use, dietary quality score, physical activity, and total body fat percentage

## Discussion

The primary finding in the present study was that a 2-year change in skeletal muscle mass index in addition to its baseline status was associated with the diabetes occurrence in a community-based population. To the best of our knowledge, this is the first study to investigate the association between changes in muscle mass and the risk of diabetes incidence in a general Chinese population.

These findings emphasize the importance of early detection and control of muscle mass loss for the prevention of diabetes. An increased risk of diabetes is not only associated with low muscle mass but can also develop silently in the face with increasing muscle mass loss.

Previous studies revealed that aged skeletal muscle with decreased oxidative capacity led to altered mitochondrial biogenesis, which may be impaired by age-dependent accumulations of point mutations in human mitochondrial (mt) DNA in addition to pro-inflammatory processes [14, 15]. Both mitochondrial dysfunction and chronic low-grade inflammation are associated with insulin resistance. Although β-cell failure is the sine qua non for development of type 2 diabetes, skeletal muscle insulin resistance is considered to be the initiating or primary defect that is evident decades before β-cell failure and overt hyperglycemia develops [16–19]. It is likely that significantly lower skeletal muscle mass results in reduced capacity for glucose disposal, but few prospective studies have investigated the risk of incident diabetes in adults with sarcopenia.

The present study demonstrated that baseline skeletal muscle mass index was an independent predictor of future diabetes incidence in adults with impaired glucose regulation; however, the said relationship was not found in adults with normal glucose tolerance probably due to the fewer metabolic risk factors in the population, and it might manifest with a relatively longer follow-up. This is consistent with previous findings from two population-based prospective studies conducted in America [20, 21]. One study concerned that men with insulin-resistance in the highest quartile had higher odds of 5% or more loss of total lean mass and appendicular lean mass than those in the lowest quartile [20]. The other study found that greater muscle area was associated with a lower risk of diabetes for older women with normal weight [21]. However, muscle mass is fundamentally correlated with body size, indicating that subjects with a larger body size may have larger muscle mass; therefore, when evaluating the adequacy of muscle mass, skeletal muscle mass index has been used. Besides, few similar cohort studies have been conducted among the Asian populations. Only one Korean study conducted in 6895 middle-aged and older individuals found that low muscle mass index was an independent risk factor for type 2 diabetes [10].

The rate of muscle loss with age appears to be fairly consistent, approximately 1%–2% per year past the age of 50 years. This study demonstrated that the loss rate significantly higher than natural loss in muscle mass had an over 2-fold risk of developing diabetes after adjusting for baseline skeletal muscle mass index and other risk factors in adults with normal glucose tolerance and impaired glucose regulation. Additionally, a reduced risk of developing diabetes was accompanied by an increase in skeletal muscle mass index regardless of whether the population's baseline level was high or below average. This finding suggests that the short-term effect of a change in skeletal muscle mass index on diabetes occurrence is not less than the effect of its baseline status that may represent a long-term effect on diabetes. It is not too late to initiate control of muscle mass loss, even in adults with prediabetes.

Our study has several limitations. First, magnetic resonance imaging is the gold standard for the measurement of skeletal muscle mass; however, it is not a convenient method for assessing skeletal muscle mass in a relatively large population-based study. Regarding the accuracy of BIA in the assessment, a previous study found that the correlation coefficient between BIA and magnetic resonance imaging was 0.93 and the standard error of the estimate for predicting skeletal muscle mass from BIA was 9%, which suggested a reasonable estimation in our study [22, 23]. Second, our results were limited to a single ethnic group. Since there may be relatively large differences in body composition even within Asian populations, it is difficult to generalize our findings to larger populations. Third, we found that an increase in skeletal muscle mass had no effect on the transformation from impaired glucose regulation to normal glucose tolerance. Ideally, further studies should extend follow-up time to verify the result.

## Conclusions

In conclusion, our study demonstrated that in addition to baseline low muscle mass, 2-year change in skeletal muscle mass was also associated with the incidence of diabetes. The risk of diabetes occurrence is not only dependent on the presence of low muscle mass but also by the loss of muscle mass.

## Data Availability

The dataset analyzed during the current study is available from the corresponding author upon reasonable request.
